# Stringent thresholds in SARS-CoV-2 IgG assays lead to under-detection of mild infections

**DOI:** 10.1186/s12879-021-05878-2

**Published:** 2021-02-18

**Authors:** David W. Eyre, Sheila F. Lumley, Denise O’Donnell, Nicole E. Stoesser, Philippa C. Matthews, Alison Howarth, Stephanie B. Hatch, Brian D. Marsden, Stuart Cox, Tim James, Richard J. Cornall, David I. Stuart, Gavin Screaton, Daniel Ebner, Derrick W. Crook, Christopher P. Conlon, Katie Jeffery, Timothy M. Walker, Timothy E. A. Peto

**Affiliations:** 1 Big Data Institute, Nuffield Department of Population Health, University of Oxford, Oxford, UK; 2 Oxford University Hospitals NHS Foundation Trust, Oxford, UK; 3 NIHR Oxford Biomedical Research Centre, University of Oxford, Oxford, UK; 4 NIHR Health Protection Research Unit in Healthcare Associated Infections and Antimicrobial Resistance at University of Oxford in partnership with Public Health England, Oxford, UK; 5 Microbiology Department, John Radcliffe Hospital, Headley Way, Oxford, OX3 9DU UK; 6 Nuffield Department of Medicine, University of Oxford, Oxford, UK; 7 Kennedy Institute of Rheumatology Research, University of Oxford, Oxford, UK; 8 Target Discovery Institute, University of Oxford, Oxford, UK; 9 Oxford University Clinical Research Unit, Ho Chi Minh City, Vietnam

**Keywords:** SARS-CoV-2, COVID-19, Serology, Antibodies, Anosmia, Ageusia

## Abstract

**Background:**

Thresholds for SARS-CoV-2 antibody assays have typically been determined using samples from symptomatic, often hospitalised, patients. In this setting the sensitivity and specificity of the best performing assays can both exceed 98%. However, antibody assay performance following mild infection is less clear.

**Methods:**

We assessed quantitative IgG responses in a cohort of healthcare workers in Oxford, UK, with a high pre-test probability of Covid-19, in particular the 991/11,475(8.6%) who reported loss of smell/taste. We use anosmia/ageusia and other risk factors as probes for Covid-19 infection potentially undiagnosed by immunoassays by investigating their relationship with antibody readings either side of assay thresholds.

**Results:**

The proportion of healthcare workers reporting anosmia/ageusia increased at antibody readings below diagnostic thresholds using an in-house ELISA (*n* = 9324) and the Abbott Architect chemiluminescent microparticle immunoassay (CMIA; *n* = 11,324): 426/906 (47%) reported anosmia/ageusia with a positive ELISA, 59/449 (13.1%) with high-negative and 326/7969 (4.1%) with low-negative readings. Similarly, by CMIA, 518/1093 (47.4%) with a positive result reported anosmia/ageusia, 106/686 (15.5%) with a high-negative and 358/9563 (3.7%) with a low-negative result. Adjusting for the proportion of staff reporting anosmia/ageusia suggests the sensitivity of both assays in mild infection is lower than previously reported: Oxford ELISA 89.8% (95%CI 86.6–92.8%) and Abbott CMIA 79.3% (75.9–82.7%).

**Conclusion:**

Following mild SARS-CoV-2 infection 10–30% of individuals may have negative immunoassay results. While lowered diagnostic thresholds may result in unacceptable specificity, our findings have implications for epidemiological analyses and result interpretation in individuals with a high pre-test probability. Samples from mild PCR-confirmed infections should be included in SARS-CoV-2 immunoassay evaluations.

**Supplementary Information:**

The online version contains supplementary material available at 10.1186/s12879-021-05878-2.

## Background

Serological tests for antibodies to SARS-CoV-2, the virus that causes Covid-19, have been used to estimate the extent of Covid-19 exposure in national [1, 2] and regional populations, as well as in subgroups of interest including healthcare workers (HCWs) [3, 4]. Additionally, antibody results may be informative about risk of future infection, at least in the short term [5].

One key element of deploying antibody testing is determining assay thresholds for confirming the detection of antibodies. Typically, this has been done based on collections of pre-pandemic sera (i.e. known negative samples) and samples from patients with PCR-confirmed Covid-19 (deemed highly likely to be antibody positive) [6–8]. Often, these ‘known positive’ sera have been derived from individuals who accessed PCR diagnostic testing based on the presence of clinical symptoms, with a bias towards those with severe enough symptoms to present to hospital where diagnostic testing was concentrated early in the pandemic. As a result, it is not clear how applicable antibody assay thresholds are to those with prior asymptomatic or mild infection which may not have met syndromic criteria for testing, such as presence of fever. Furthermore, even in symptomatic individuals, those with milder disease may have lower viral loads [9, 10] and therefore be more likely to be falsely PCR-negative and omitted from assay calibration cohorts. Limited numbers of studies have looked specifically at those with asymptomatic or mild infection [6], however lower IgG titres have been reported in patients with milder infection compared to those admitted to ICU [11] and one study of 26 PCR-positive asymptomatic individuals showed detectable IgG in only 4 (15%) a median of 29 days post PCR [12], in contrast another showed neutralising antibodies in 47/48 (98%) HCWs with mild infection by 28–41 days [13].

We have recently undertaken SARS-CoV-2 serological testing in a large cohort of UK HCWs [3]. In keeping with other researchers [14–16], we reported that loss of smell (anosmia) or taste (ageusia) were highly predictive of Covid-19 [3].Additionally, several HCWs tested were antibody-negative despite clinical syndromes consistent with Covid-19, including household contacts of PCR-confirmed cases. Therefore, here we use the presence of anosmia/ageusia along with other risk factors for Covid-19 as probes for Covid-19 infection in our HCW cohort. We use these to search for Covid-19 that was potentially undiagnosed by immunoassays by investigating their relationship with antibody readings either side of the assay threshold.

## Methods

### Setting, participants and immunoassays

Asymptomatic HCWs from across 4 teaching hospitals in Oxfordshire, UK, were invited to participate in voluntary staff testing for Covid-19 by nasal and oropharyngeal swab PCR and serological testing. The cohort and associated methods have been previously described in detail [3]. Prior to testing staff were asked to provide questionnaire data on symptoms since 01 February 2020. Additionally, PCR results were available for staff who were tested following symptoms (predominantly fever or new persistent cough), which in the majority of cases were mild; only 10 staff required hospital admission. Therefore, the large majority of staff with immunological evidence of previous infection had only mild or no previous symptoms.

Serology for SARS-CoV-2 IgG to nucleocapsid protein was performed using the Abbott Architect i2000 chemiluminescent microparticle immunoassay (CMIA; Abbott, Maidenhead, UK). Samples were also tested by a high-throughput enzyme-linked immunosorbent assay (ELISA) developed at the University of Oxford detecting antibodies to trimeric spike antigen [8, 17, 18].

### Analysis

The first sample tested per individual was analysed. Immunoassay readings were compared with the proportion of staff reporting anosmia or ageusia, as well as other symptoms. We also considered the association of different antibody readings with risk factors for Covid-19, including living with a person with PCR-confirmed Covid-19, after adjusting for other Covid-19 exposures, role, specialty area worked in and ethnicity [3]. We also compared antibody readings with the predicted probability of Covid-19 from the same previously described multivariable regression model.

To examine if anosmia/ageusia were found across all individuals in our cohort, we investigated if age, gender, ethnicity, specialty area worked in or role affected the likelihood of reporting anosmia/ageusia given an individual was subsequently shown to be SARS-CoV-2 IgG positive. Univariable and multivariable logistic regression models were fitted, using natural cubic splines to account for non-linearity of continuous predictors, choosing the best fitting model on backwards selection and number of spline knots using AIC values.

The previously defined and manufacture’s thresholds for confirming detection of anti-SARS-CoV-2 IgG are 8 million and 1.4 arbitrary units for the Oxford ELISA and Abbott CMIA respectively. We used the distribution of the proportion of staff reporting loss of smell/taste within varying antibody reading groups to define a range of readings which we considered “high negative” (equivocal) (see Results, 4.0–7.9 million for the Oxford ELISA and 0.20–1.39 for the Abbott CMIA). We used the proportion of individuals with “low negative” antibody readings (Oxford ELISA < 4 million, Abbott CMIA < 0.2) reporting loss of smell or taste to estimate the background rate of anosmia/ageusia unrelated to Covid-19. We subtracted this background proportion (*P*_*baseline*_) from the proportion of individuals reporting anosmia/ageusia in those with high negative (potentially equivocal) antibody results (*P*_*eq*_) and the proportion with positive antibody results (*P*_*pos*_) to estimate the overall proportion of anosmia/ageusia attributable to Covid-19. This allowed us to estimate adjusted sensitivity (*S*_*adj*_) of the Oxford and Abbott immunoassays, compared with the previously reported sensitivity (*S*), accounting for the excess of anosmia/ageusia in individuals with elevated but negative antibody results. We denote the number of individuals with positive antibody results *N*_*pos*_ and the number with high negative results *N*_*eq*_ such that:

$$ {S}_{adj}=S\ast {N}_{pos}\left({P}_{pos}-{P}_{baseline}\right)/\left({N}_{pos}\left({P}_{pos}-{P}_{baseline}\right)+{N}_{eq}\ \left({P}_{eq}-{P}_{baseline}\right)\right) $$

Bootstrapping with 1000 iterations was used to estimate the uncertainty in adjusted sensitivity results, accounting for variation in previously reported sensitivity as well as variation arising from the proportions from the current analysis. We also undertook a sensitivity analysis assuming a proportion of the anosmia/ageusia in those with low negative antibody readings was due to Covid-19.

All analyses were undertaken using R version 3.6.3. Exact binomial confidence intervals are presented for proportions.

### Ethics

Asymptomatic staff data collection and testing were part of enhanced hospital infection prevention and control measures instituted by the UK Department of Health and Social Care. Staff provided informed consent as part of an online sign-up process. For the purposes of this study existing deidentified data from staff testing were obtained from the Infections in Oxfordshire Research Database (IORD) which has generic Research Ethics Committee (South Central - Oxford C Research Ethics Committee; 19/SC/0403), Health Research Authority and Confidentiality Advisory Group approvals (19/CAG/0144). Use of data from IORD does not require individual consent, however details of an opt-out procedure are made publicly available. This study was conducted in accordance with relevant guidelines and regulations.

## Results

Eleven thousand four hundred seventy-seven hospital staff provided a first serum sample between 23rd April and 20th August 2020, of whom 11,475 provided complete associated symptom and risk factor survey data. The median (IQR) age of staff was 39 (30–50) years, 8463 (74%) were female. Detailed breakdowns of the occupational roles and specialities of staff have been provided previously [3], the largest groups included nurses and healthcare assistants (4383, 38%), doctors (1746, 15%), administrative staff (1384, 12%) and therapists and other allied health professionals (1054, 9%). Within the 11,475 serum samples obtained, 9324 were analysed using an anti-spike ELISA developed in Oxford and 906 (9.7%) had IgG detected. Eleven thousand three hundred forty-two samples were analysed using the Abbott Architect CMIA and 1093 (9.6%) had anti-nucleocapsid IgG detected. Within 9191 samples tested by both platforms, 788 (8.6%) had IgG detected by both Abbott CMIA and Oxford ELISA, 114 (1.2%) by only Abbott CMIA and 106 (1.2%) by the Oxford ELISA only.

### Antibody readings in individuals with a prior PCR-positive nasopharyngeal swab

**Fig. 1:**
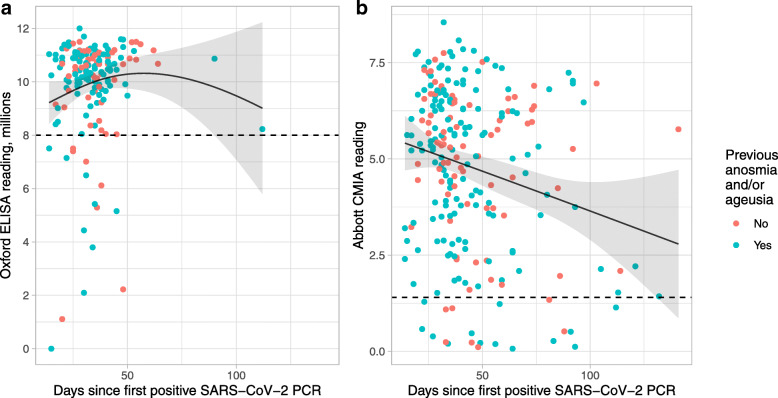
SARS-CoV-2 IgG antibody readings in 245 convalescent symptomatic healthcare workers ≥ 14 days following a positive PCR test. Panel **a** shows readings using the Oxford ELISA assay targeting trimeric spike protein (*n* = 171) and panel **b** shows readings using the Abbott CMIA targeting nucleocapsid protein (*n* = 240). The dashed horizontal lines show the pre-defined threshold for reporting antibody detection (Oxford ELISA 8 million, Abbott CMIA 1.4). The solid line and ribbon shows the fitted mean value and 95% confidence interval using a linear regression model with a 3 knot spline. Points are coloured by whether staff reported previous anosmia (loss of smell) and/or ageusia (loss of taste) since 01 February 2020 when asked prior to serological testing: 159/245 staff (65%) reported anosmia/ageusia

Two hundred forty-five staff participating in asymptomatic testing had previously tested PCR-positive for SARS-CoV-2 on a combined nasal and oropharyngeal swab. These PCR results were obtained as part of testing offered to symptomatic staff and were taken ≥14 days prior to serological testing. The median (IQR) [range] time interval between the first PCR-positive swab and serological testing was 37 (30–52) [14–141] days. Not all 245 staff were tested using both immunoassays: 155/171 (90.6, 95%CI 85.2–94.6%) were IgG positive using the Oxford ELISA when tested between 14 and 112 days after their PCR test (Fig. 1a) and 220/240 (91.7, 95%CI 87.4–94.8%) using the Abbott CMIA after 14 to 141 days (Fig. 1b).

For most staff with a negative antibody result, this could not be fully explained by waning titres, given the relatively short time since their first positive PCR test and the overall trend in readings (Fig. 1). There was no evidence that staff testing IgG negative were more likely to have an elevated Charlson comorbidity score or have attended a haematology, oncology or rheumatology clinic (as a proxy for immunosuppression) since 01 January 2019 (Supplementary Figure S1).

### Relationship between anosmia/ageusia and antibody readings

As only a minority of the HCWs studied had a prior positive PCR test (245/11,475, 2.1%), and these individuals had all been sufficiently symptomatic to access the test (fever ≥37.8 °C or a new persistent cough), we investigated other less stringent clinical markers of possible Covid-19. A total of 991/11,475 (8.6%) staff reported loss of smell/taste. In those with anosmia/ageusia who could recall a date of symptom onset, the large majority, 801/811 (99%), underwent serum sampling for serology ≥14 days after their symptom onset; a median (IQR) 40 (38–70) days later.

**Fig. 2:**
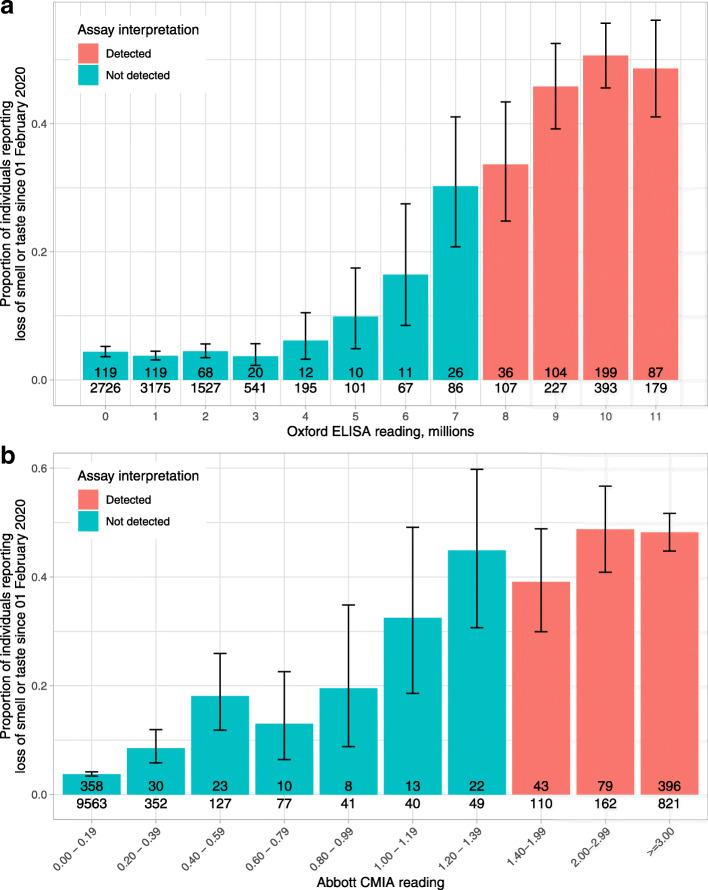
Proportion of staff with anosmia or loss of taste by antibody reading. Panel **a** shows the results using a trimeric spike ELISA and panel **b** the results from the Abbott CMIA targeting nucleocapsid protein, with blue showing results called negative and red showing those called as positive based on pre-defined assay thresholds. The number of individuals with these symptoms is shown in each bar, and the total number of individuals with each antibody reading below the bar. The error bars show 95% confidence intervals. For the Oxford ELISA readings each value is rounded down, such that for example a value of 1.7 million is within the 1 million bar

The proportion of staff reporting loss of smell/taste increased at antibody readings below the diagnostic threshold for both the Oxford ELISA (Fig. 2a) and Abbott CMIA (Fig. 2b), consistent with possible Covid-19 in those classified as having high negative (equivocal) antibody readings. The diagnostic threshold for detection of antibody by the Oxford ELISA is 8 million. However, from readings of 4 million and above the proportion of staff reporting loss of smell/taste started to rise from a baseline of ~ 4 to 30% at 7 million (0–3 million vs. 4 million: exact *p* = 0.15 and vs. 5 million: *p* = 0.009). Similarly, Abbott CMIA readings of 0.2 and above (diagnostic threshold 1.4) were associated with increased loss of smell/taste (< 0.2 vs. 0.2–0.39: *p* < 0.001). Similar trends, albeit with less power in some instances, were seen for antibody readings and self-reported fever, myalgia and new persistent cough (Supplementary Figures S2, S3 and S4).

**Fig. 3:**
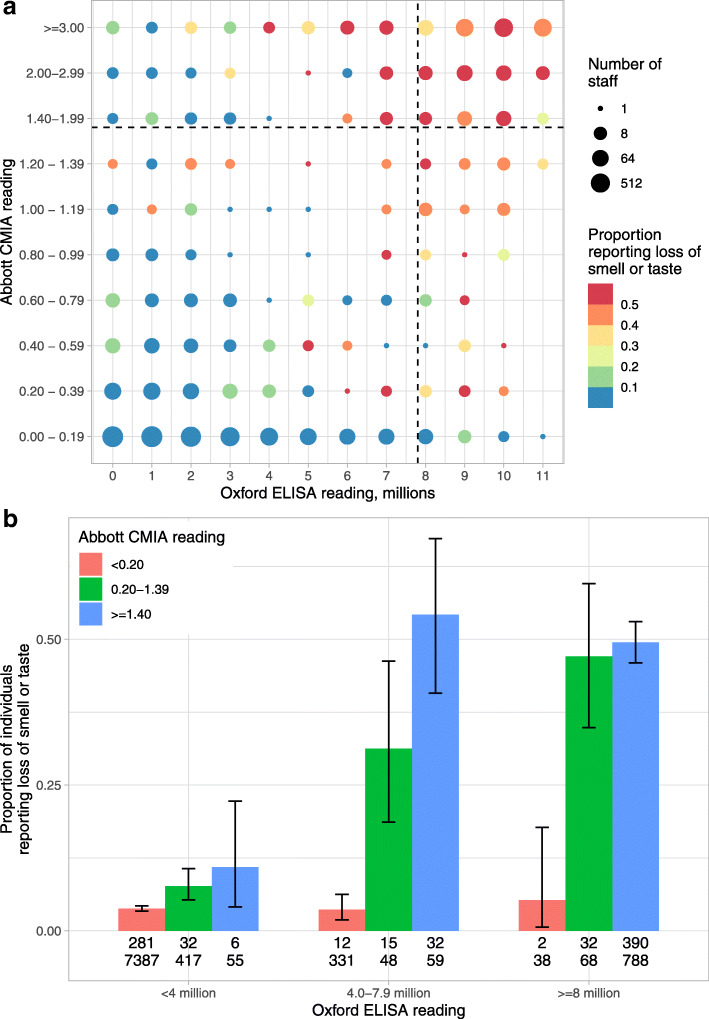
The relationship between Abbott CMIA and Oxford ELISA readings and loss of smell or taste in 9191 samples tested by both platforms. Panel **a** compares the number of individuals with combinations of Abbott CMIA and Oxford ELISA readings, the size of each circle represents the number of individuals and the colour the proportion reporting loss of smell or taste. Panel **b** groups the data by positive, high-negative (equivocal) and low-negative readings for both assays, the numbers shown beneath each bar are the number of individuals reporting loss of smell/taste and the total number of individuals with the antibody reading

Regarding Oxford ELISA readings between 4 and 8 million and Abbott CMIA readings between 0.2 and 1.4 as equivocal, equivocal results by one assay were frequently associated with positive results by the other (Fig. 3a). However, we also observed an increase in anosmia/ageusia in staff with equivocal Abbott readings where Oxford ELISA results were equivocal or low negative. Whereas, the incidence of anosmia/ageusia was near baseline with low negative Abbott readings irrespective of the Oxford ELISA reading (Fig. 3b).

### Impact on estimated serology sensitivity for Covid-19

To estimate the extent to which current antibody thresholds may under-estimate past Covid-19 infections we considered the percentage of individuals reporting loss of smell/taste by 3 antibody reading categories (Table 1). Conservatively, considering those with an Oxford ELISA reading of < 4 million and reporting loss of smell/taste as the background rate of anosmia/ageusia unrelated to Covid-19, an estimated additional 9.0% of individuals with antibody readings of 4.0–7.9 million reported Covid-19-associated anosmia/ageusia, and 42.9% with readings of > 8 million. Assuming that the sensitivity of antibody tests is similar in those reporting and not reporting loss of smell/taste and taking the previously reported sensitivity of the Oxford ELISA as 99.1% (531/536) [8], the sensitivity in those with mild infection was estimated to be 89.8% (95%CI 86.6–92.8%). However, more individuals with a previous positive PCR and low antibody readings reported anosmia than would be expected by a background rate of 4.1% (Fig. 1). Therefore, with an alternative example assumption that the background rate of anosmia/ageusia is 3%, i.e. some of the anosmia/ageusia in those with low antibody readings is Covid-19-related, the sensitivity would be 75.6% (95%CI 68.3–82.0%). For the Abbott CMIA with a previously reported sensitivity of 92.7% (90.2–94.8%) [8], the estimated sensitivity following mild/asymptomatic infection was 79.3% (75.9–82.7%) after adjustment for a background rate of 3.7% (Table 1) and 70.5% (67.6–81.1%) assuming a 3% background rate of anosmia/ageusia.

**Table 1:** Assessment of additional potential SARS-CoV-2 infections using loss of smell/taste and Oxford ELISA readings (*n* = 9324 samples) and Abbott CMIA readings (*n* = 11,342). The previously reported sensitivity of the Oxford ELISA is 99.1% (95%CI 97.8–99.7%) and the Abbott CMIA is 92.7% (90.2–94.8%) [8]

	**Oxford ELISA reading**		
	**< 4 million** **Low negative**	**4.0–7.9 million** **High negative**	**≥8 million** **Positive**
**Loss of smell/taste reported**	326 (4.1%)	59 (13.1%)	426 (47.0%)
**Loss of smell/taste not reported**	7643 (95.9%)	390 (86.9%)	480 (53.0%)
**Total**	7969	449	906
**Additional estimated number with loss of smell/taste above baseline**	–	40 (9.0%)	389 (42.9%)
	**Abbott CMIA reading**		
	**< 0.2** **Low negative**	**0.2–1.39** **High negative**	**≥1.4** **Positive**
**Loss of smell/taste reported**	358 (3.7%)	106 (15.5%)	518 (47.4%)
**Loss of smell/taste not reported**	9205 (96.3%)	580 (84.5%)	575 (52.6%)
**Total**	9563	686	1093
**Additional estimated number with loss of smell/taste above baseline**	–	81 (11.8%)	478 (43.7%)

### Predictors of anosmia and ageusia in seropositive HCWs

We investigated if loss of smell or taste was as a generalisable marker for Covid-19 to assess the extent to which our findings from those reporting anosmia/ageusia might apply to all individuals with mild Covid-19. We considered associations between anosmia/ageusia in SARS-CoV-2 IgG seropositive staff and demographic and occupational factors (Supplementary Table S1). Of the 1211 staff with SARS-CoV-2 IgG antibodies detected by either the Oxford ELISA or Abbott CMIA, 554 (46%) reported loss of smell and/or taste since 01 February 2020 when asked before serum sampling. There was no association with self-reported ethnicity. Male gender (adjusted odds ratio 0.72 (95%CI 0.50–0.89) and occupational role, but not specialty or age were selected in a multivariable model (Supplementary Table S1). Overall, besides gender, broadly similar rates of anosmia/ageusia were seen in most groups with Covid-19.

### Association between Covid-19 risk factors and antibody readings

**Fig. 4:**
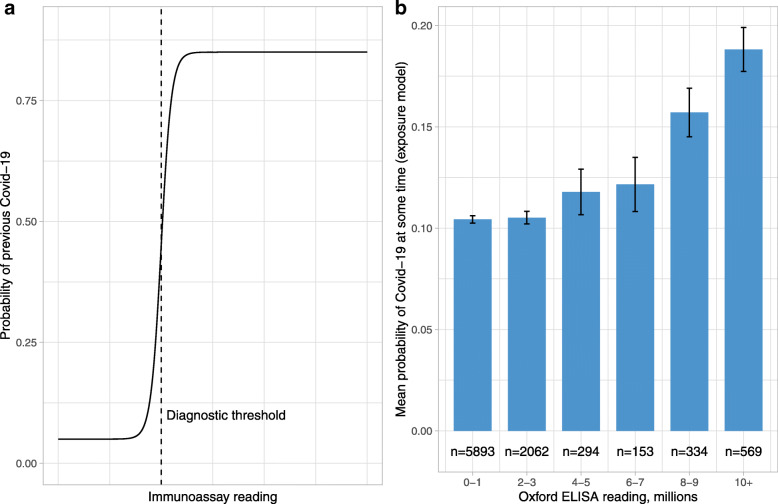
Ideal (panel **a**) and actual (panel **b**) relationship between the probability of Covid-19 and antibody reading. The probability of Covid-19 in panel B was generated from a multivariable model containing risk factors including Covid-19 exposures in the community and at work, ethnicity, healthcare worker role and specialty area worked in [3]

We also considered the relationship between exposure risk factors and antibody readings. Figure 4a shows an ideal assay where low readings are associated with low probability of Covid-19 and increases in assay readings at the diagnostic threshold result in a rapid switch to a high probability of Covid-19. Figure 4b shows the actual relationship obtained in 9305 staff members with an Oxford ELISA result and a previously generated mean probability of Covid-19 at some time using a model based on healthcare and community Covid-19 exposures, ethnicity, healthcare role, and specialty described in [3]. The probability of Covid-19 assigned by the model to those with ELISA readings of 0–1 million was 10.4%, which was similar to those with readings of 2–3 million but rose to 11.8% (t-test *p* = 0.02 vs. 0–1 million) by 4–5 million and 12.2% by 6–7 million (*p* = 0.01). A similar trend in point estimates was seen when considering only the greatest risk factor for Covid-19, household contact with a PCR-confirmed case, although the number of individuals with this exposure was less, limiting certainty (Supplementary Figure S5).

## Discussion

More individuals have had Covid-19 than are identified using immunoassays calibrated on PCR-positive cases enriched for hospitalised patients. We show, using two different immunoassays that target different SARS-CoV-2 antigens, that intermediate assay results, below diagnostic thresholds for positivity, are associated with increased rates of anosmia, ageusia and other symptoms, as well as being more frequent in individuals with a higher risk of Covid-19. Additionally, we show that around 1 in 10 previously symptomatic and PCR-positive HCWs had negative antibody assays when tested a median (IQR) 37 (30–52) days following a first positive PCR test.

We used self-reported anosmia and ageusia rates across antibody readings to estimate how many mild/asymptomatic Covid-19 cases might be missed by current assay thresholds. Although these were subjective and retrospectively reported symptoms, we observed a clear relationship with antibody readings (Fig. 2). Adjusting for rates of anosmia/ageusia in those with high negative (equivocal) antibody readings produces an estimated test sensitivity for the Oxford ELISA of 89.8% (95%CI 86.6–92.8%) rather than the previously reported 99.1% (97.8–99.7%). Similarly, the adjusted Abbott CMIA sensitivity is 79.3% (75.9–82.7%) compared to 92.7% (90.2–94.8%) [8]. However, we observed several individuals reporting anosmia/ageusia with a positive PCR result but negative antibody results, including low negative readings (Fig. 1). Therefore, as a sensitivity analysis we calculated the adjusted sensitivity assuming an example background rate of anosmia/ageusia of 3%, which lead to estimated sensitivities for the Oxford ELISA and Abbott CMIA following mild/asymptomatic infection of 75.6% (95%CI 68.3–82.0%) and 70.5% (67.6–81.1%) respectively. This example background rate is plausible as self-reported rates of anosmia in previous studies vary, e.g. from ~ 1 to 5%, with rates of 3% in the ages typical of the HCWs studied in one series [19]. However, these are studies of established objective anosmia, subjective rates may differ, and the question used for our study asked only about new onset loss of smell or taste since 01 February 2020, i.e. excluded established anosmia. It should also be noted that we analyse antibody readings from assays designed for qualitative use, and so these readings may not change linearly with changes in underlying antibody responses.

Studies of closed communities or households can also be used to put a lower bound on test sensitivity if universal exposure is considered likely. For example, seroprevalence on the US Navy ship the USS Theodore Roosevelt was 60% following a large outbreak, demonstrating immunoassay sensitivity must be at least this amount (a further 6% were PCR-positive, but antibody negative, possibly reflecting sampling shortly after Covid-19 onset before antibody levels could rise) [14].

Our analysis aims to estimate assay performance in mild infections at current diagnostic thresholds, rather than to propose new thresholds. As such, the specificity of each assay is that previously reported, i.e., ≥99% for both assays [8]. However, if assay thresholds were lowered to improve sensitivity in mild infection, this would also result in reduced specificity. For example, in our dataset 449/9324 (4.8%) of Oxford ELISA results fell within the high-negative (equivocal) range we defined, as did 686/11342 (6.0%) of Abbott results. If the majority of these individuals are uninfected, then using this lower threshold specificity might be expected to fall to < 95%, i.e. unacceptably low in low prevalence settings. As such it may not be possible to detect all previous mild infections serologically while maintaining adequate specificity. Future studies of PCR-confirmed mild infection and pre-pandemic samples are needed to provide sufficient data to propose any adjustment to assay thresholds. In populations with a high pre-test probability of infection, the underlying prevalence may be sufficiently high that lower specificity can be tolerated, and in this setting considering reporting equivocal results, which may or may not prompt testing on second assay may be helpful.

The main limitation of our study is the lack of a PCR test in all HCWs reporting anosmia. We cannot say what proportion of those with equivocal antibody responses and anosmia/ageusia would have had a positive PCR test if tested shortly after infection. Furthermore, given the anosmia/ageusia were self-reported and may have had other causes, our study is limited by the lack of a pre-pandemic control group asked the same question regarding new onset loss of taste or smell between February and May of a previous year. There is therefore uncertainty about the background rate of anosmia/ageusia, and the attributability of this to other respiratory viral causes in this context [20, 21]. Reported anosmia/ageusia may also vary between settings and populations, for example less anosmia is reported in East Asian patients with Covid-19 [22], however we found similar rates of anosmia/ageusia in staff from Asian ethnic groups (predominantly south and south-east Asian) with Covid-19 to staff identifying as white. Additionally, some antibody responses may have been missed, although 99% of all those reporting anosmia/ageusia and providing a date of symptom onset were tested ≥14 days after symptom onset and before antibody responses began to fall substantially. Follow up studies are needed to evaluate the extent to which individuals with symptoms or exposures strongly suggestive of Covid-19 such as anosmia or a PCR-confirmed household contact, but negative antibody results, have other evidence of infection, for example from T cell assays and also to assess the extent of neutralising antibody activity across a range of antibody readings. Specific Covid-19 T cell responses have been reported in seronegative individuals who have been exposed to SARS-CoV-2 [23], including in HCWs from our own setting [24].

Antibody results can be used for multiple applications, including epidemiological and modelling studies. The sensitivity of SARS-CoV-2 serology in those with mild symptoms, i.e. the majority of the infected population, is likely to be lower than previously reported, 90% or less, which is likely to have implications for epidemiological modelling and forecasting. Antibody results may also inform assessments of the risk of an individual being re-infected. However, for individuals with a high pre-test probability of Covid-19, negative serology does not exclude previous infection and possible protective immunity.

## Conclusions

Following mild SARS-CoV-2 infection 10–30% of individuals may have negative immunoassay results. While negative SARS-CoV-2 immunoassays following mild infection may not be unexpected, here we are able to quantify that this may be more common than previously appreciated. Our data also highlight, that in contrast to the approach used in evaluations to date [6–8], samples from individuals with mild and asymptomatic infection should be included in SARS-CoV-2 immunoassay evaluations in sufficient numbers to assess sensitivity in different populations. Our findings have important implications for epidemiological studies and individuals interpreting their antibody results.

## Supplementary Information

**Additional file 1.**

## Data Availability

The datasets analysed during the current study are not publicly available as they contain personal data but are available from the Infections in Oxfordshire Research Database (https://oxfordbrc.nihr.ac.uk/research-themes-overview/antimicrobial-resistance-and-modernising-microbiology/infections-in-oxfordshire-research-database-iord/), subject to an application and research proposal meeting the ethical and governance requirements of the Database. For further details on how to apply for access to the data and for a research proposal template please email iord@ndm.ox.ac.uk.

## Abbreviation

HCW: Healthcare worker
