# Supplementary material for: Stringent thresholds in SARS-CoV-2 IgG assays lead to under-detection of mild infections
Source: BMC Infect Dis. 2021 Feb 18;21:187. doi: 10.1186/s12879-021-05878-2 (PMC7889711; doi:10.1186/s12879-021-05878-2)
Supplement: Supplementary file 1 — Additional file 1. [file 12879_2021_5878_MOESM1_ESM.pdf]

Stringent thresholds in SARS-CoV-2 IgG assays lead to under-detection of mild infections: Supplement

*David W Eyre[1,2,3,4], Sheila F Lumley[2,5], Denise O'Donnell[5], Nicole E Stoesser[2,3,4,5], Philippa C Matthews[2,3,5], Alison Howarth[5], Stephanie B Hatch[5], Brian D Marsden[5,6], Stuart Cox[2], Tim James[2], Richard J Cornall,[5] David I Stuart[5], Gavin Screaton[5], Daniel Ebner[5,7], Derrick W Crook[2,3,5], Christopher P Conlon[2,5], Katie Jeffery[2], Timothy M Walker[2,5,8] Timothy EA Peto[2,3,5]

[1]Big Data Institute, Nuffield Department of Population Health, University of Oxford, Oxford, UK

[2]Oxford University Hospitals NHS Foundation Trust, Oxford, UK

[3]NIHR Oxford Biomedical Research Centre, University of Oxford, Oxford, UK

[4]NIHR Health Protection Research Unit in Healthcare Associated Infections and Antimicrobial Resistance at University of Oxford in partnership with Public Health England, Oxford, UK

[5]Nuffield Department of Medicine, University of Oxford, Oxford, UK

[6]Kennedy Institute of Rheumatology Research, University of Oxford, UK

[7]Target Discovery Institute, University of Oxford, Oxford, UK

[8]Oxford University Clinical Research Unit, Ho Chi Minh City, Vietnam

*Corresponding author:

David Eyre, david.eyre@bdi.ox.ac.uk,

+44 (0)1865 221081,

Microbiology Department, John Radcliffe Hospital, Headley Way, Oxford, OX3 9DU

Supplementary figures and table

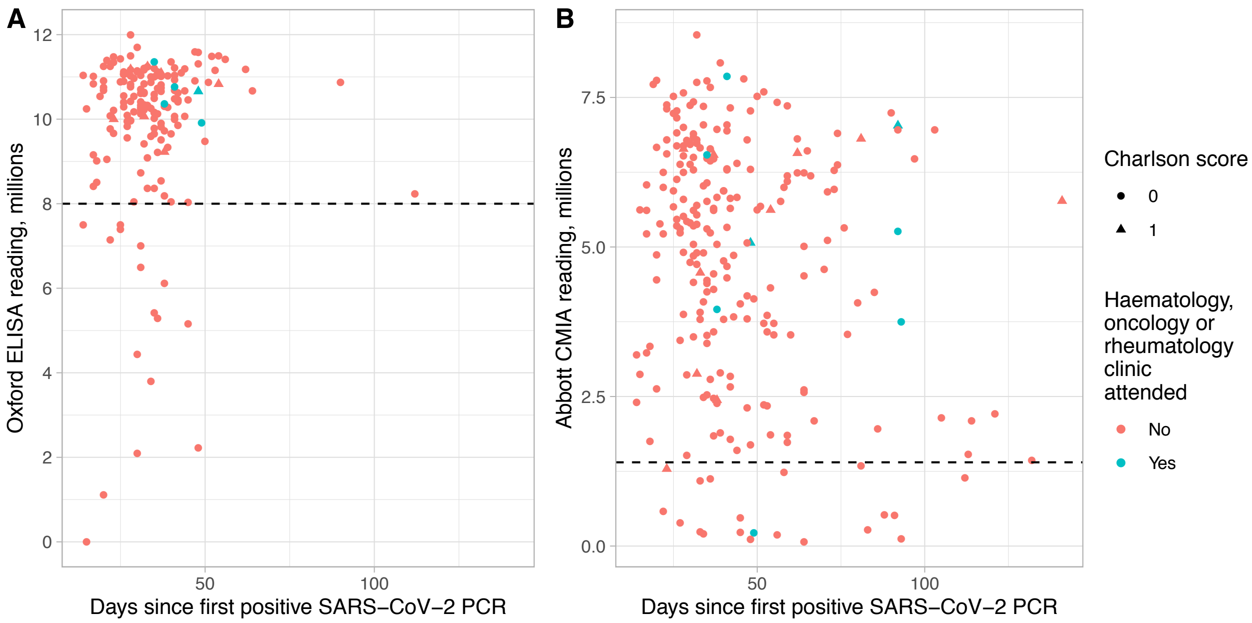

Supplementary Figure S1. SARS-CoV-2 IgG antibody readings in 245 convalescent symptomatic healthcare workers ≥14 days following a positive PCR test*. Panel A shows readings using the Oxford ELISA assay targeting trimeric spike protein (n=171) and panel B shows readings using the Abbott CMIA targeting nucleocapsid protein (n=240). The dashed horizontal lines show the threshold for reporting antibodies as detected. Points are coloured by whether staff had attended a haematology, oncology or rheumatology clinic since 01 January 2019 (as a proxy for immunosuppression), and the plotted shapes indicate Charlson comorbidity scores based on diagnostic codes from any admissions to hospital since 01 January 2019.*

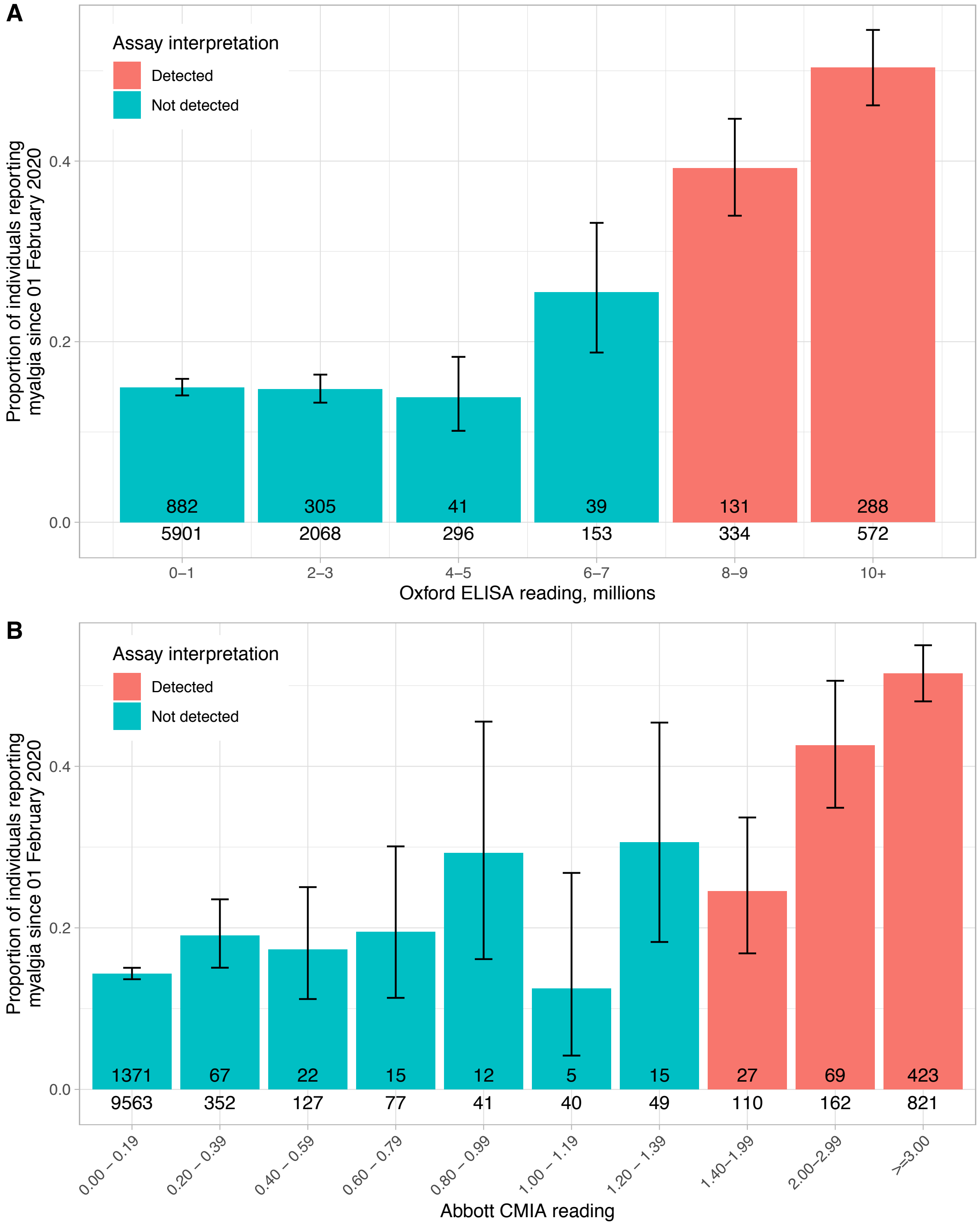

Supplementary Figure S2. Association of previous myalgia with immunoassay reading.
Panel A shows the results using a trimeric spike ELISA and panel B the results from the Abbott CMIA targeting nucleocapsid protein, with blue showing results called negative and red showing those called as positive based on pre-defined assay thresholds. The number of individuals with myalgia is shown in each bar, and the total number of individuals with each antibody reading below the bar. The error bars show 95% confidence intervals. For the Oxford ELISA readings each value is rounded down, such that for example a value of 1.7 million is within the 1 million bar.

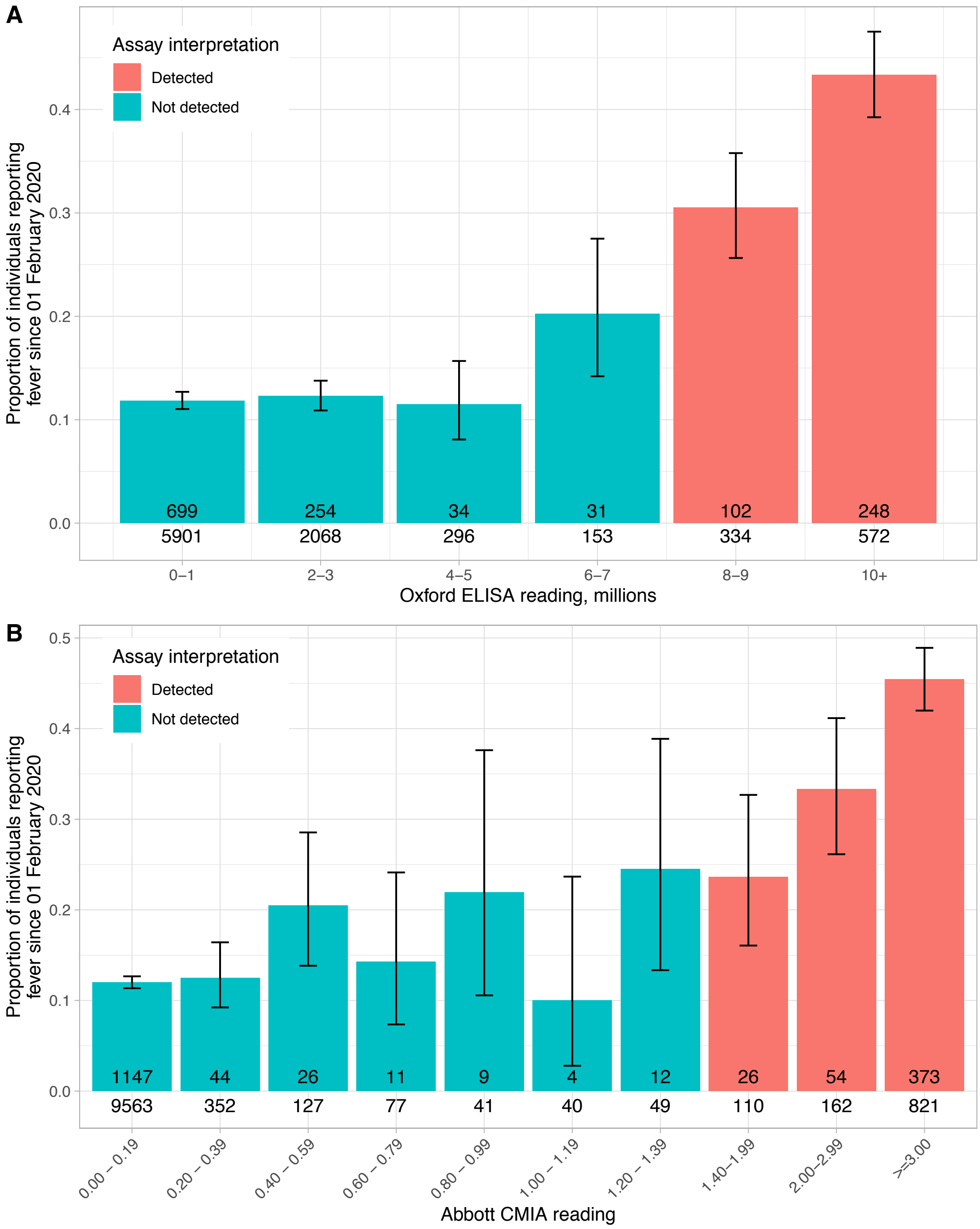

Supplementary Figure S3. Association of previous fever with immunoassay reading.
Panel A shows the results using a trimeric spike ELISA and panel B the results from the Abbott CMIA targeting nucleocapsid protein, with blue showing results called negative and red showing those called as positive based on pre-defined assay thresholds. The number of individuals with previous fever is shown in each bar, and the total number of individuals with each antibody reading below the bar. The error bars show 95% confidence intervals. For the Oxford ELISA readings each value is rounded down, such that for example a value of 1.7 million is within the 1 million bar.

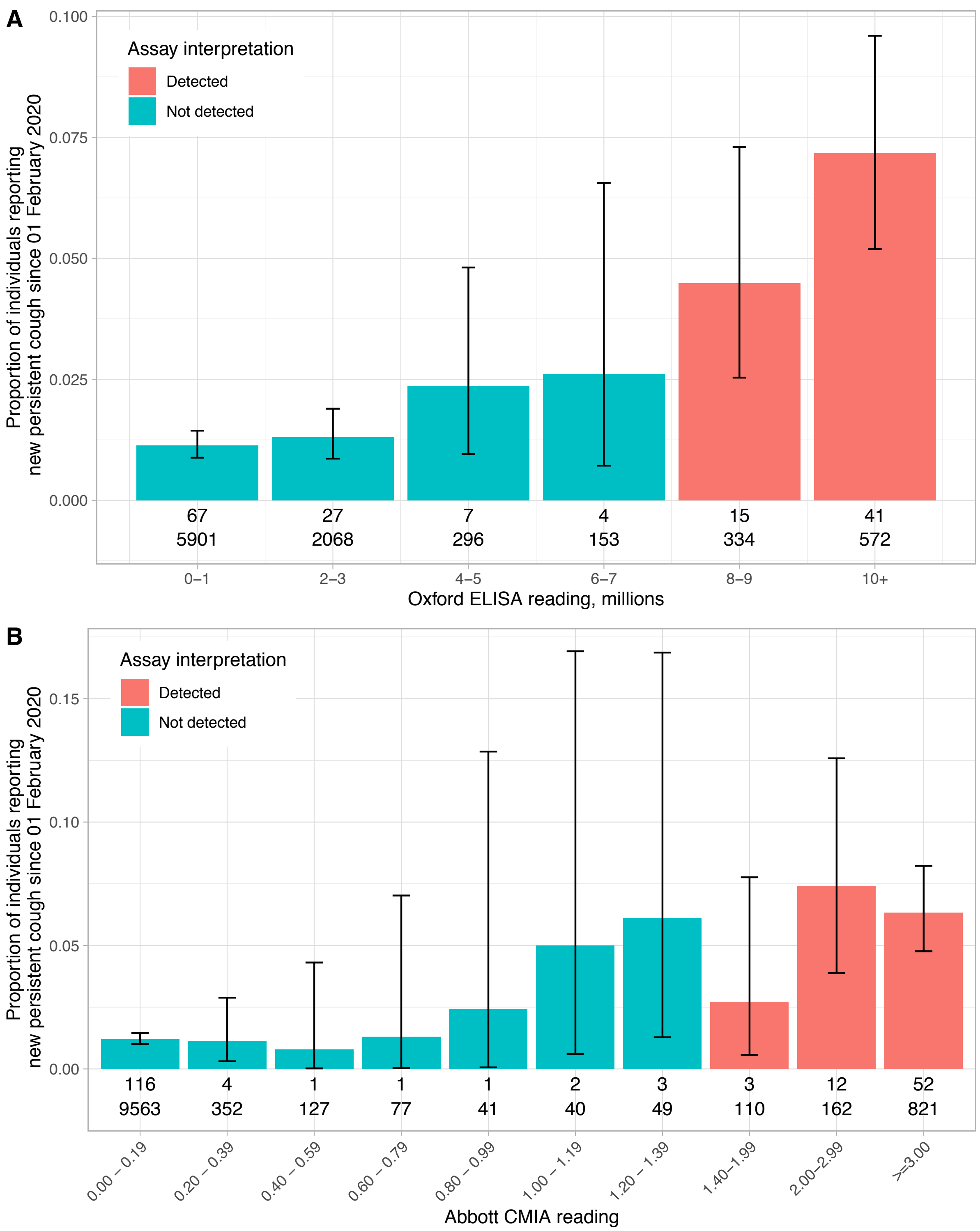

Supplementary Figure S4. Association of previous new persistent cough with immunoassay reading.

Panel A shows the results using a trimeric spike ELISA and panel B the results from the Abbott CMIA targeting nucleocapsid protein, with blue showing results called negative and red showing those called as positive based on pre-defined assay thresholds. The number of individuals with previous new persistent cough is shown in each bar, and the total number of individuals with each antibody reading below the bar. The error bars show 95% confidence intervals. For the Oxford ELISA readings each value is rounded down, such that for example a value of 1.7 million is within the 1 million bar.

		Total	Number reporting anosmia	Percentage with Covid-19 reporting anosmia/ ageusia	Univariable odds ratio for anosmia/ ageusia	95% CI	p value	Multivariable odds ratio for anosmia	95% CI	p value
Ethnicity	White	720	333	46%	1.00					
	Asian	300	141	47%	1.03	(0.79, 1.35)	0.83			
	Black	81	32	40%	0.76	(0.47, 1.21)	0.25			
	Chinese	10	5	50%	1.16	(0.33, 4.05)	0.81			
	Mixed	36	15	42%	0.83	(0.42, 1.64)	0.59			
	Not stated	17	7	41%	0.81	(0.31, 2.16)	0.68			
	Other	47	21	45%	0.94	(0.52, 1.70)	0.83			
Role	Administrative staff	94	39	41%	1.00			1.00		
	Biomedical scientist and laboratory staff	35	23	66%	2.70	(1.20, 6.07)	0.02	2.81	(1.24, 6.34)	0.01
	Senior doctor	63	24	38%	0.87	(0.45, 1.67)	0.67	1.01	(0.51, 1.97)	0.99
	Junior doctor	128	68	53%	1.60	(0.93, 2.74)	0.09	1.77	(1.02, 3.08)	0.04
	Nurse, healthcare assistant	599	285	48%	1.28	(0.82, 1.99)	0.27	1.27	(0.82, 1.98)	0.29
	Other	112	46	41%	0.98	(0.56, 1.72)	0.95	1.05	(0.60, 1.84)	0.87
	Other allied health professional	47	24	51%	1.47	(0.73, 2.97)	0.28	1.49	(0.73, 3.01)	0.27
	Porter, Domestic	62	15	24%	0.45	(0.22, 0.92)	0.03	0.54	(0.26, 1.13)	0.10
	Physiotherapists, Occupational therapists, Speech and language therapists	45	19	42%	1.03	(0.50, 2.12)	0.94	1.01	(0.49, 2.09)	0.97
	Security, Estates, Catering	26	11	42%	1.03	(0.43, 2.49)	0.94	1.22	(0.50, 3.00)	0.66
Speciality area	Other	406	172	42%	1.00					
	Anaesthetics	21	8	38%	0.84	(0.34, 2.06)	0.70			
	Emergency Medicine	44	22	50%	1.36	(0.73, 2.54)	0.33			
	General Surgery, Urology, Plastics, Vascular, Cardiothoracic surgery	83	41	49%	1.33	(0.83, 2.13)	0.24			
	Haematology, Oncology	53	22	42%	0.97	(0.54, 1.73)	0.91			
	Infectious Diseases, Respiratory	30	11	37%	0.79	(0.37, 1.70)	0.54			
	Intensive Care Medicine	48	23	48%	1.25	(0.69, 2.28)	0.46			

	Medicine	228	117	51%	1.43	(1.04, 1.99)	0.03			
	Obstetrics and Gynaecology	18	8	44%	1.09	(0.42, 2.82)	0.86			
	Ophthalmology, Ear, Nose and Throat, Maxillofacial surgery	10	8	80%	5.44	(1.14, 25.95)	0.03			
	Paediatrics	45	28	62%	2.24	(1.19, 4.22)	0.01			
	Radiology	35	18	51%	1.44	(0.72, 2.88)	0.30			
	Specialist Medicine	130	53	41%	0.94	(0.63, 1.40)	0.75			
	Trauma and Orthopaedics, Rheumatology	60	23	38%	0.85	(0.48, 1.48)	0.56			
Gender	Female	861	418	49%	1.00			1.00		
	Male	347	135	39%	0.65	(0.52, 0.87)	0.002	0.72	(0.50, 0.89)	0.02
	Prefer not to say	2	1	50%	1.00	(0.07, 17.00)	0.97	1.15	(0.07, 18.88)	0.92
	Trans	1	0	0%	0.00	(0.00, >100)	0.97	0.00	(0.00, >100)	0.97
Age	per 10 year increase				0.91	(0.82, 1.00)	0.05			

Supplementary Table S1. Association between demographic and workplace factors and self-reported anosmia or ageusia in 1211 healthcare workers with positive SARS-CoV-2 serology.

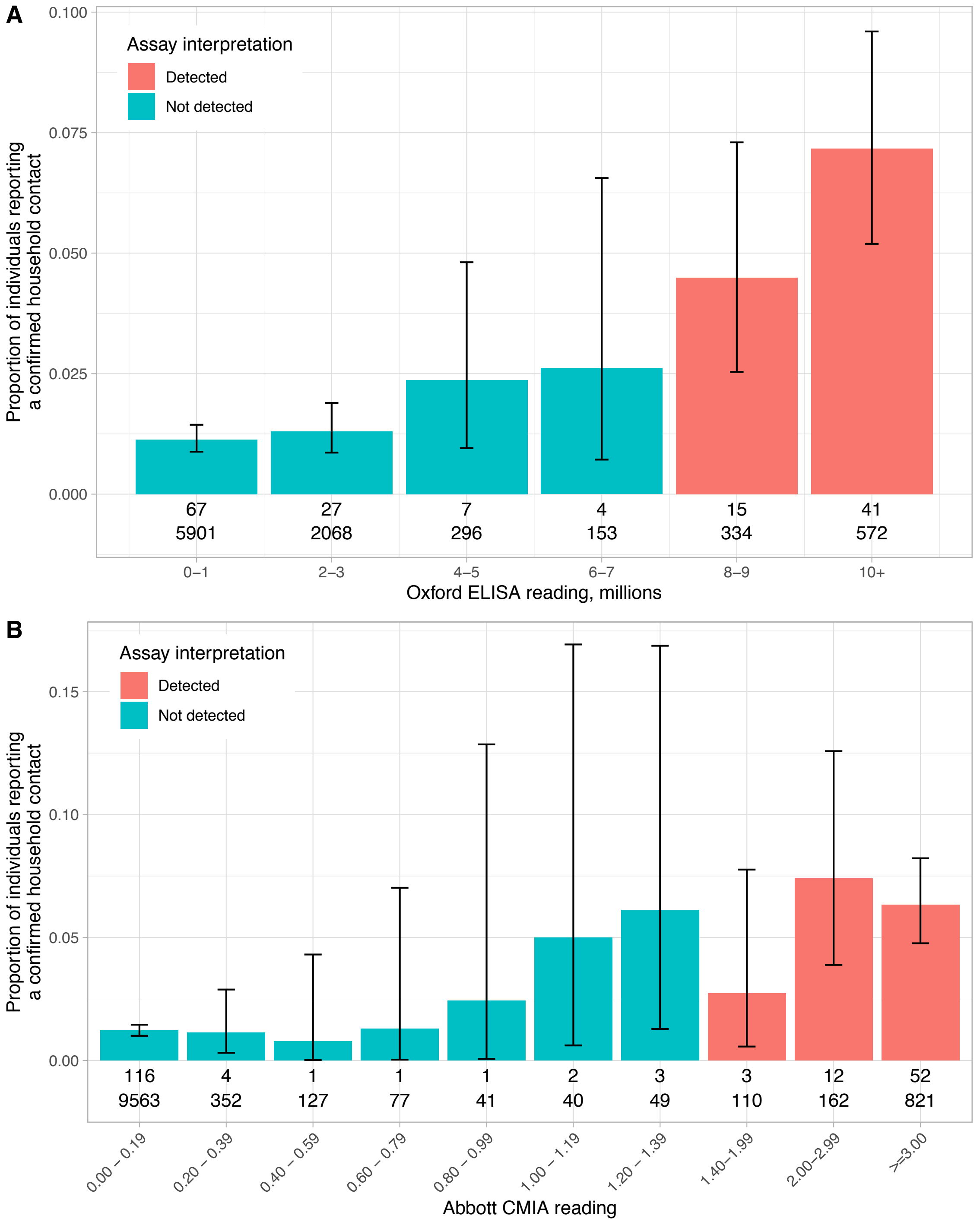

Supplementary Figure S5. Association of a PCR-confirmed household contact with immunoassay reading. *Panel A shows the results using a trimeric spike ELISA and panel B the results from the Abbott CMIA targeting nucleocapsid protein, with blue showing results called negative and red showing those called as positive based on pre-defined assay thresholds. The number of individuals with a household contact is show just below each bar, and the total number of individuals with each antibody reading below that. The error bars show 95% confidence intervals. For the Oxford ELISA readings each value is rounded down, such that for example a value of 1.7 million is within the 1 million bar.*
